# Mr. Clayton, on Ching's Worm Lozenges

**Published:** 1807-02

**Authors:** 


### *To the Editors of the Medical and Physical Journal.*

Gentlemen,

FOR the information of Mr. Fowler, I beg leave to transmit you the following extract from one of the hand-bills, circulated by the father of the unfortunate child, who died in consequence of taking Ching's Worm Lozenges.

"A patent quack medicine, known by the name of Ching's Worm Lozenges, having been lately intruded on, and recommended to the public, I deem it a duty incumbent on me to declare, that the above worm lozenges, from the quantity of mercury contained in them, are a most destructive structive and deadly poison, less active, but equally capable of destroying life, as arsenic.—Numerous, it is to be feared, are the cases where life has been destroyed by them, and the cause not suspected, from the want of medical assistance. But the following lamentable case, which has happened in my *own family*, can be well attested by many professional gentlemen, and which is confirmed by an inquest taken on view of the body, before W. W. Boulton, Esq. coroner for this place, on Tuesday the 3d inst. will, it is hoped, operate as a caution to parents and others, who have the care of children, and prevent the administering a sure mercurial poison in the form of Ching's Lozenges, from which the most direful effects may be apprehended.

"On Sunday and Wednesday the 4th and 7th December, 1803, Ching's Worm Lozenges were administered, according to the directions, to my unfortunate child, (a fine boy of three years old) and on Friday the 9th, he was in a high state of salivation. Medical assistance was immediately called in, when he was pronounced in imminent danger from mercurial lozenges. Remedies were immediately applied, and all the aid that medicines could afford resorted to, but without effect; for the mouth ulcerated, the teeth dropped out, the hands contracted, and a complaint was made of a pricking pain in them and the feet, the body became flushed and spotted, and at last black; convulsions succeeded, attended with a slight delirium; and a mortification destroyed the face, which proceeding to the brain put a period (after indescribable torments) to the life of the little sufferer, on Sunday the 1st inst. twenty-eight days after he had taken the poisonous lozenges. This shews how cautious people ought to be in administering quack medicines.

"A coroner's inquest being summoned, and the evidence of the medical gentlemen adduced, the Jury returned a verdict of "Poisoned by Ching's Worm Lozenges."

"To Messrs. John and James Sagner, who paid an unremitting attention to my unfortunate child, and were the first medical gentlemen called in, I beg leave to return my thanks for the assistance afforded; and also to Dr. John Alderson, and the other professional gentlemen; convinced that every thing was done, which skill and experience could dictate, though unhappily without the desired effect.

"THOMAS CLAYTON."

*Hull, January 9th*, 1804.

Mr. Clayton further adds, that "Mr. Ching declined all communication, though informed of the above case in its first first stage." Mr. C. appears to have been ignorant of Mr. Ching's demise, which took place previous to the date of the case. He was a regular country practitioner of some celebrity, and from the successful exhibition of his lozenges in private practice, was induced to advertize them. With the result of this undertaking the public are sufficiently acquainted.

Much might be said on this subject, were it not for infringing on the limits of a Journal, whose pages delight the lovers of free enquiry. It is to be hoped that Dr. Harrison and his co-adjutors, in their zeal for medical reform, will not lose sight of this important object, nor be appaled by the formidable barrier which presents itself in the shape of *stamp duties*.

I am, &c.

